# Profiling changes in cortical astroglial cells following chronic stress

**DOI:** 10.1038/s41386-018-0105-x

**Published:** 2018-05-29

**Authors:** Stephanie Simard, Gianfilippo Coppola, Christopher A. Rudyk, Shawn Hayley, Robyn J. McQuaid, Natalina Salmaso

**Affiliations:** 1 Department of Neuroscience, Carleton University, Ottawa, ON Canada; 2 Child Study Center, Yale University, New Haven, CT USA; 3 The Royal Ottawa Hospital, Ottawa, ON Canada

## Abstract

Recent studies have suggested that cortical astroglia play an important role in depressive-like behaviors. Potential astroglial contributions have been proposed based on their known neuroplastic functions, such as glutamate recycling and synaptic plasticity. However, the specific mechanisms by which astroglial cells may contribute or protect against a depressive phenotype remain unknown. To delineate astroglial changes that accompany depressive-like behavior, we used astroglial-specific bacTRAP mice exposed to chronic variable stress (CVS) and profiled the astroglial translatome using translating ribosome affinity purification (TRAP) in conjunction with RNAseq. As expected, CVS significantly increased anxiety- and depressive-like behaviors and corticosterone levels and decreased GFAP expression in astroglia, although this did not reflect a change in the total number of astroglial cells. TRAPseq results showed that CVS decreased genes associated with astroglial plasticity: RhoGTPases, growth factor signaling, and transcription regulation, and increased genes associated with the formation of extracellular matrices such as perineuronal nets (PNNs). PNNs inhibit neuroplasticity and astroglia contribute to the formation, organization, and maintenance of PNNs. To validate our TRAPseq findings, we showed an increase in PNNs following CVS. Degradation of PNNs in the prefrontal cortex of mice exposed to CVS reversed the CVS-induced behavioral phenotype in the forced swim test. These data lend further support to the neuroplasticity hypothesis of depressive behaviors and, in particular, extend this hypothesis beyond neuronal plasticity to include an overall decrease in genes associated with cortical astroglial plasticity following CVS. Further studies will be needed to assess the antidepressant potential of directly targeting astroglial cell function in models of depression.

## Introduction

Depression is among the leading causes of disability worldwide [1], with an estimated 350 million people affected. Currently, treatments for depression include psychotherapy, antidepressant medications, and in some extreme cases, electroconvulsive therapy; however, an estimated 30–40% of individuals are treatment resistant [2]. Several theories, such as the monoaminergic hypothesis, have been used to explain the neurobiological basis of mood disorders; however, these fail to resolve key findings, including considerable lags between monoamine level changes and symptom relief. Accumulating evidence suggests that disturbances of plasticity, including dendritic arborization, neurogenesis, and synaptic organization, underlie the etiology and successful treatment of depression [3, 4].

### Astrocytes and depression

Astroglial cells outnumber neurons by up to 50 times in the brain and are essential to neuronal integrity and functionality. Recent evidence suggests that, like neurons, astroglial cells include several subtypes that differ in function and show distinct protein expression patterns. These include protoplasmic astrocytes, known for their typical star-shaped pattern; reactive astrocytes; and astroglial stem cells that exhibit a radial-like morphology. Throughout the lifespan, astroglia play pivotal roles in metabolizing neurotransmitters, modifying cell connectivity, and providing trophic support to neurons; as neural stem cells, astroglia give rise to astrocytes, oligodendrocytes, and neurons both under basal conditions and in response to injury [5, 6].

Several studies have investigated a role for glial cells in the etiology and treatment of depression [7–13, 14, 15]. Postmortem studies from patients with major depressive disorder have consistently shown a decreased density of glial cells in the prefrontal cortex (PFC) [16, 17]. Similarly, rats that are subjected to chronic variable stress (CVS), an animal model for depression, show impairments in cortical glial function and gliogenesis. Exposure to various stressors induced a 20% decrease in glial fibrillary acidic protein (GFAP), an intermediate filament protein expressed in a subset of astrocytes in adulthood [13]. Perhaps the most striking support for astroglial involvement in depressive behaviors is that glial ablation and not neural ablation within the PFC was sufficient to induce depressive-like behaviors similar to those seen after CVS [13].

A recent review postulates that antidepressants activate astrocytes, priming them to carry out specific functions that result in the reactivation of cortical plasticity and readjustment of abnormal neuronal networks (Czeh et al., 2013). For example, antidepressants affect numerous astrocytic functions, including the availability of various neurotransmitters (e.g., serotonin, glutamate, or GABA), the regulation of energy homeostasis, control over the blood–brain barrier integrity [13], regulation of gap-junction proteins and synaptic plasticity [18], and the release of neurotrophic factors [19]. Collectively, these studies indicate significant changes in glia in the depressed brain, in particular within the amygdala, hippocampus, and PFC. However, the nature and therapeutic relevance of these changes remains largely unknown and most of the work to date has focused on hippocampal and amydalar changes, despite an obvious role for the cortex in cognitive processes underlying depressive symptomatology.

In the current study, we fully characterized astroglial responses to stress in order to elucidate the mechanisms by which astroglial cells are involved in the pathophysiology of depression. We employed a CVS paradigm and profiled the astroglial translatome using translating ribosome affinity purification (TRAP) [20–22] in conjunction with RNAseq (TRAPseq). We used immunohistochemistry to validate these changes and reversed stress-induced depressive behaviors through the degradation of stress-induced changes in perineuronal nets (PNNs).

## Methods

### Experimental animals and procedure

Forty-eight adult male C57/BL6- AldHL1-L10GFP transgenic mice (Breeding Colony, Carleton University, Ottawa) or wild-type littermates were single housed in standard (27 × 17 × 13 cm^3^) fully transparent polypropylene cages. Twenty-four animals were placed in a stress room, while the remaining half were placed in a shared control room (Refer to supplemental methods for animal groups). Mice were placed in each room a minimum of 48 h prior to the first day of the experiment in order to acclimate. Basic cage enrichment was provided (i.e., nesting material and shelter). The mice were maintained on a 12-h light/dark cycle in a temperature controlled (21 °C) environment with ad libitum access to food and water (except when overnight fasts formed part of CVS). All animal use procedures were approved by the Carleton University Committee for Animal Care, according to the guidelines set by the Canadian Council for the Use and Care of Animals in Research.

### Chronic variable stress

**Fig. 1:**
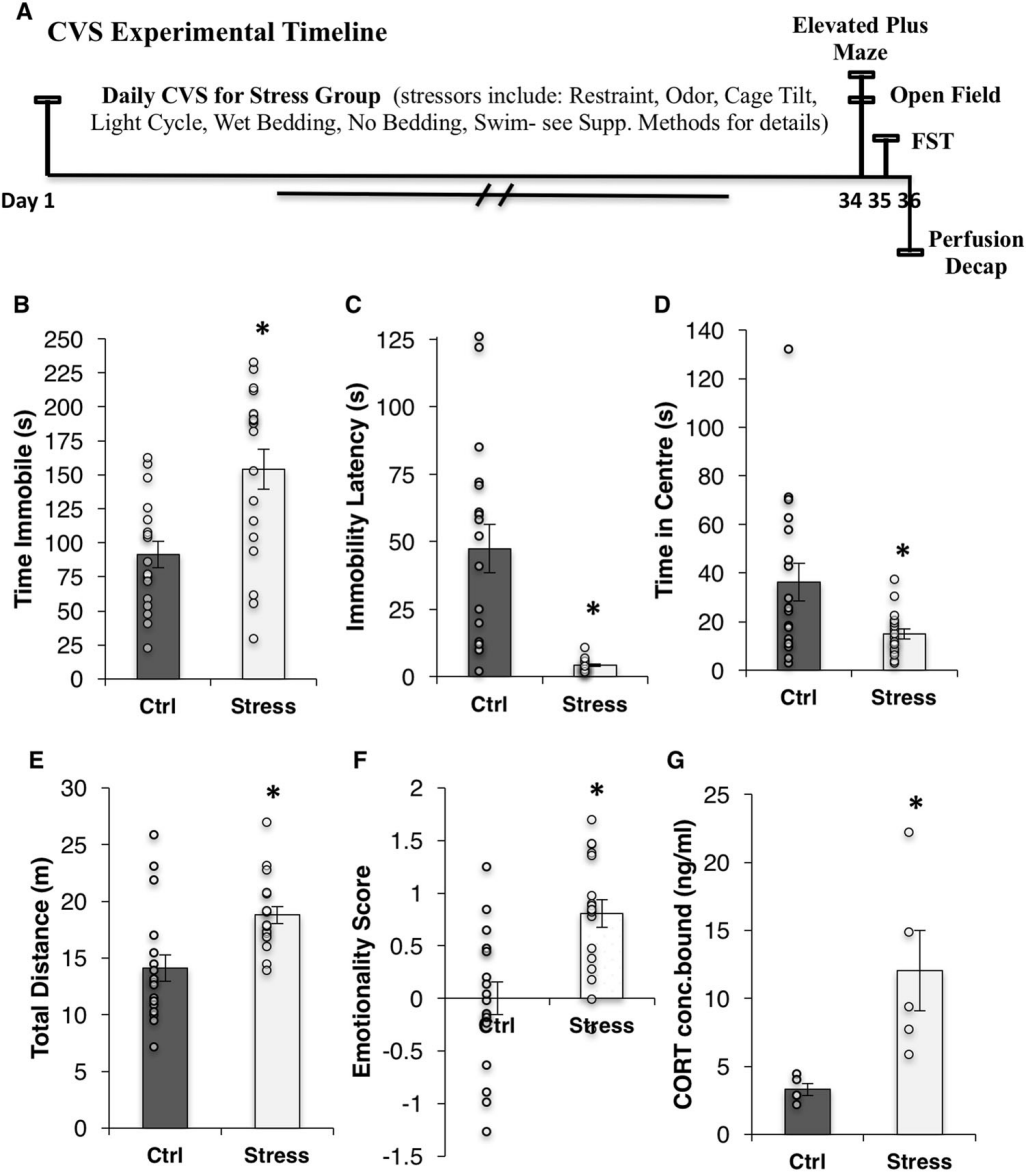
CVS reliably induces depressive- and anxiety-like phenotype. **a** CVS experimental timeline. **b**, **c** Forced swim test: total time immobile (**b**) and the latency to immobility (**c**). **d**, **e** Open field test: total time in center (**d**) and total distance traveled in the entire field (**e**). **f** Emotionality Score ([28], see supplemental methods for detailed calculations). **g** Basal serum corticosterone levels as measure by ELISA. Bars denote group mean and error bars denote SEM. *p ≤ 0.01; data points reflect individual scores

This experiment used a CVS paradigm adapted from the Duman laboratory [7] to induce a depressive-like behavioral phenotype (see supplemental methods for stress schedule). The animals in the stress group were exposed to various mild stressors daily over 35 days (2–3 per 24-h period) that were uncontrollable in both time and duration to prevent habituation. This long-term exposure allowed the investigation of neuroadaptations that result from chronic stress (see Fig. 1a for timeline).

### Behavioral tests

All behavioral tests were as per our previously published work [23, 24], please see supplemental methods for further details.

### Tissue harvesting and immunohistochemical analyses

Upon completion of the experiment, 36 animals were euthanized and tissue harvested for immunohistochemistry. Half of the animals were intraperitoneally injected with 100 mg/kg of sodium pentobarbital and intracardially perfused with phosphate-buffered saline (PBS) followed by 4% paraformaldehyde. Brains were harvested and post-fixed in 4% paraformaldehyde overnight followed by 48 h in a 30% sucrose solution at 4 °C. Brains were kept at −80 °C and sectioned on a ThermoFisher cryostat at 30 μm and adhered to electrostatic slides in alternation, totaling 25 sister section slides, each representing an entire brain. For immunohistochemistry, sections were blocked in PBS containing 0.3% Triton (PBS-T) with 10% horse serum and subsequently incubated in primary antibody overnight and then washed thoroughly and incubated in a fluorescein-conjugated secondary antibody (see supplemental methods for antibody table).

### Corticosterone enzyme-linked immunosorbent assay (ELISA)

Ten green fluorescent protein-negative (GFP−) mice (5 CTRL, 5 Stress) were rapid-decapitated and blood samples were collected in EDTA-coated Eppendorf tubes and centrifuged for approximately 10 min at 4 °C. The serum was collected and stored at −80 °C until processing. Corticosterone levels were determined by ELISA using the Assay Design Kit (Corticosterone #900–097, Lot# D1260724) and quantified on a microplate reader set to 405 nm and to 570–590 nm for correction. Quantifications were normalized to standards and outliers removed using the MyAssays.com automated software.

### Surgical procedures

Forty-four, wild-type C57/B6 mice (Charles River, Saint Constant, Quebec) were exposed to CVS (or control) for 32 days and, on the following day, injected with Chondroitinase ABC (ChABC) (or vehicle control) bilaterally into the PFC in order to degrade the PNNs. See supplemental methods for further details.

### Cell counting and microscopic analyses

Unbiased estimates of total cell number/hemisphere were obtained via a Zeiss AxioImager M2 motorized fluorescent microscope with ApoTome (Carl Zeiss, Thornwood, NY, USA) attached to a motorized stage and connected to a computer running the StereoInvestigator Software^TM^ (MicroBrightfield, Colchester, VT, USA). Serial coronal sections (one every 30 µm) were used for all counts. Contours of the entire cortex were drawn. Cells were counted for the expression of individual and/or co-expressed markers using the optical fractionator probe with a 40× objective. Tri-dimensional sampling boxes with 3 out of the 6 exclusion borders were automatically placed by StereoInvestigator at each grid intersection point [25, 26]. Two sizes were employed: 150 × 150 × 30 µm^3^ for abundant cell types (GFP, GFAP, glutamine synthetase (GS), Vimentin) and 300 × 300 × 30 µm^3^ for the less-abundant PNNs (minimum 200 cells/cortex counted). The total number of cells for one hemisphere of the entire cortex are reported.

Confocal acquisition of images for co-localized proteins were taken using Zeiss Airyscan 800TM. 63×+ oil images were processed using Airyscan Processing on the ZEN software (Zeiss TM).

### Statistical analyses

Data from experiments with two groups (CTRL vs CVS) were analyzed by Student’s *T*-test, while data from experiments with two independent variables (CTRL/CVS and VEH/ChABC) were analyzed by a between-subjects factorial analysis of variance (ANOVA). If a significant interaction was found, then ANOVAs were followed by post-hoc simple comparisons with Bonferonni correction for non-orthogonal tests. Correlational analyses between numbers of GFAP, PNNs, and Vimentin+ cells with behavioral measures were conducted using Pearson product moment correlations (one-tailed). In cases where more than one predictor variable significantly correlated to a behavioral measure, a multiple regression analysis was used to determine the strongest predictor of that behavioral measure. All data were evaluated using the Stat-View (version 6.0) statistical software package available from the SAS Institute, Inc., and differences were considered statistically significant when *p* = 0.05 or less.

### TRAP followed by RNAseq

TRAP was conducted in homogenate samples of cortex obtained from AldH- EGFP-L10 control and stress mice, which express GFP in ribosomes of AldH+ cells, thus allowing for the immunoprecipitation of polysomes directly from astrocytes [20–22]. TRAP methods were conducted as previously published [27]. Twelve mice were used in total, six from each group. Briefly, mice were quickly decapitated and cortices were dissected on ice in a dissection buffer containing cyclohexamide (100 μg/ml, Sigma, dissolved in methanol, American Bioanalytical) in order to immediately freeze ribosome/mRNA (polysome) complexes. Samples were pooled (groups of two) and homogenized in extraction buffer [22] using a Teflon-glass homogenizer (Fisher #K8855100020) and then centrifuged for 10 min at 2000 × *g* at 4 °C. NP-40 (AG Scientific # P1505) and DHPC (Avanti # 850306 P) were added to the collected supernatant and incubated for 5 min on ice before centrifugation for 15 min at 20,000 × *g* until pelleted. The lysate supernatant was extracted, 30 mM DHPC was added, and then incubated with magnetic beads overnight. Magnetic beads (Streptavidin MyOne T1 Dynabeads; Invitrogen # 65601) were previously coated with Protein L (Fisher # PI-29997) for 35 min at room temperature (1 µg/µl in 1× PBS), collected on a magnetic rack (DynaMag-2; Invitrogen #123-21D), and washed 5 times in 1× PBS containing 3% protease-free bovine serum albumin (Jackson Immuno #001-000-162) and then subsequently incubated with 50 µg each of anti-GFP antibodies (HtzGFP_04 (clone19F7) and HtzGFP_02 (clone 19C8): Memorial Sloan-Kettering Monoclonal Antibody Facility) overnight at 4 °C and then washed again, prior to being incubated with the lysate. Following antibody incubation, beads were again collected on a magnetic rack, and unbound fragments were also collected and saved for RNA extraction step as total homogenate controls. Bound fragments were washed in a polysome buffer [22] and then resuspended in lysis buffer (Stratagene #400753) followed by RNA cleanup and extraction. All RNA samples were extracted using Stratagene’s Absolutely RNA Nanoprep Kit (Stratagene #400753), and unbound and bound samples were snap frozen and stored at −80 until use.

RNA samples were sent to the Yale K.E.C.K facility for sample quality control as per their standard protocols: Total RNA quality was determined by estimating the A260/A280 and A260/A230 ratios by nanodrop and RNA integrity was determined by running an Agilent Bioanalyzer gel (RNA integrity numbers ranged from 9.8 to 10). See further details of library preparation, sequencing, and TRAPseq data analyses in supplemental methods.

### Quantitative real-time PCR

To validate the enrichment of astroglial genes, input and TRAP-immunoprecipitated fragments were compared for the levels of GFAP and GS using the best-coverage TAQMAN assays (Life Technologies) and analyzed using the Applied Biosystems 7500 Real-Time PCR system and software.

## Results

### CVS induces depressive- and anxiety-like behaviors

CVS induced a significant increase in immobility time in the forced swim test (FST; *p* = 0.001) and a decrease in latency to immobility (*p* = 0.001; Fig. 1b, c). In the open field test, CVS significantly decreased the time spent in the center of the open field (*p* = 0.01; Fig. 1d); however, CVS mice were also hyperactive: they were faster (*p* = 0.002; data not shown), less immobile (*p* = 0.002; data not shown), and traveled further (*p* = 0.001) in the entire area of the open field (Fig. 1e). No differences were seen in the elevated plus maze between groups (*p* = 0.2; data not shown). In order to further demonstrate the CVS-induced phenotype, we included a total “emotionality score” as first described by the Sibille laboratory ([28], see supplemental methods for formula); a significant increase in emotionality was induced by CVS (*p* < 0.0001; Fig. 1f). Significant increases in basal corticosterone levels were also observed in response to CVS (*p* = 0.01), suggesting that overall CVS induced a reliable depressive phenotype (Fig. 1g).

### Immunohistochemical characterization of cortical astroglia following chronic stress

**Fig. 2:**
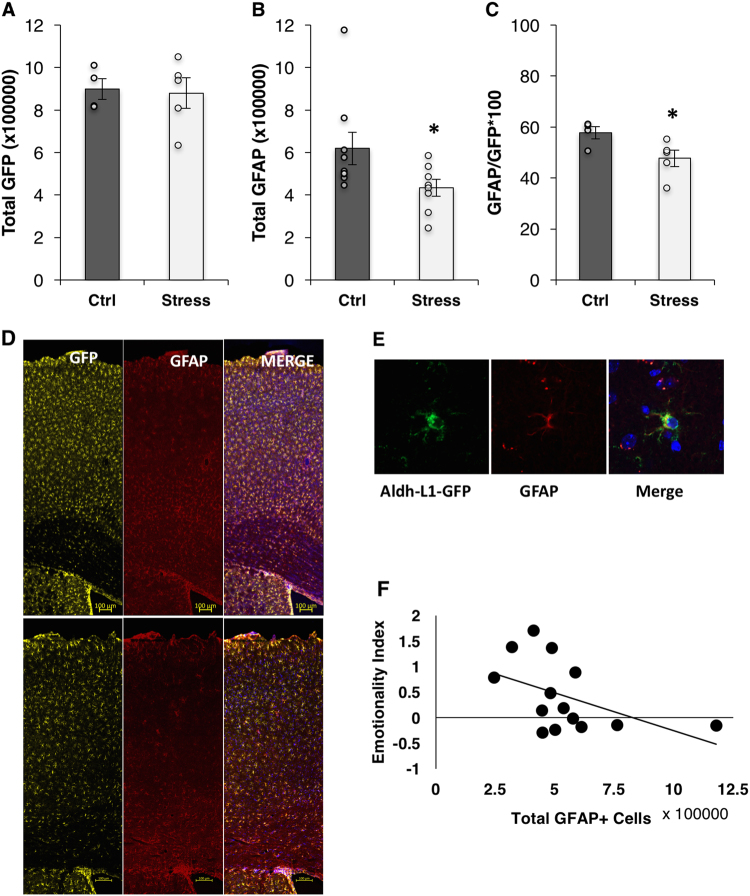
CVS does not change the number of cortical astroglial. **a** Total number of GFP+ astroglia cells per cortical hemisphere. **b** Total number of GFAP+ astroglia cells per cortical hemisphere. **c** Proportion of GFAP+ astroglia cells per cortical hemisphere. **d** Representative confocal image (20×) showing co-localization of GFAP and GFP in an astroglial cell. **e** Representative confocal image (63×) showing co-localization of GFAP and GFP in an astroglial cell. **f** Correlation scatter plot showing a negative correlation between the number of GFAP+ cells and the total emotionality score. Bars denote group mean and error bars denote SEM. *p = 0.05; data points reflect individual scores

Previous studies have shown a decrease in cortical astroglial numbers as reflected in a reduction in GFAP (an intermediate filament protein) stained cells in models of depression. We used unbiased stereology to count the total number of astroglia in the cortex of ALDH-L1-L10-GFP+ mice, which constitutively express GFP in all astroglial cells, and found that the total number of GFP+ astrocytes did not change with CVS (*p* = 0.8) (Fig. 2a, d, e). However, the total number of GFAP+ astrocytes significantly decreased with stress (*p* = 0.05; Fig. 2b, d, e). Interestingly, the proportion of GFAP+ cells represents only about half of the total number of astroglia (Fig. 2c–e). In addition, this proportion changed significantly in response to CVS, from 57 to 47%, suggesting an overall downregulation of GFAP protein in response to stress (*p* = 0.05), which is consistent with previous reports ([29], [16]). Interestingly, the number of GFAP+ cells were positively related to the center distance in the open field, *r* = 0.66, *p* = 0.005, and negatively related to the total emotionality score, *r* = −0.48, *p* = 0.04 (Fig. 2f, Table 1). No other significant relationships between GFAP and behavior were found (Table 1).

**Table 1:** Pearson correlations between GFAP, PNN, and vimentin with depressive- and anxiety-like behaviors

	^1^	^2^	^3^	^4^	^5^	^6^	^7^	^8^
1. GFAP								
2. PNN	−0.26							
3. Vimentin	−0.52*	0.45^+^						
4. Time immobile (FST)	−0.35	0.63**	.23					
5. Latency to immobility (FST)	0.25	−0.59*	−0.50*	−0.54*				
6. Time in center (OF)	0.41	−0.30	−0.14	−0.22	0.33			
7. Centre distance (OF)	0.66**	−0.16	−0.15	0.05	−0.16	0.44		
8. Time open arms (EPM)	0.34	−0.39	−0.42	−0.01	0.51*	0.35	0.23	
9. Emotionality score	−0.48*	0.69**	0.32	0.92**	−0.59*	−0.45a	−0.22	−0.07

^+^*p* = 0.05; *p < 0.05; **p < 0.01

In order to further phenotype cortical astroglia, we counted the number of Vimentin+ astroglia (vimentin is an intermediate filament protein associated with radial extensions): we found a significant increase in the number of Vimentin+ cells in the CVS group, suggesting a switch in astroglial state in response to CVS (*p* = 0.01; data not shown). Upon examining the relationships between Vimentin and behavioral measures, a significant negative relationship was found in relation to the latency to immobility, *r* = −0.50, *p* = 0.03 (Table 1).

Several studies have implicated a role for glutamatergic changes in response to stress, depressive-, and anxiety-like behaviors and subsequent therapeutic response [30, 31]. GS is an enzyme expressed in astroglia that is responsible for the breakdown of glutamate into glutamine. The number of GS+ cells did not change in response to stress (*p* = 0.6) nor did the proportion of GS+ to total astroglia (*p* = 0.5): overall approximately 60% of astroglia were GS+ (data not shown).

### Translational profiling of cortical astroglial cells

**Fig. 3:**
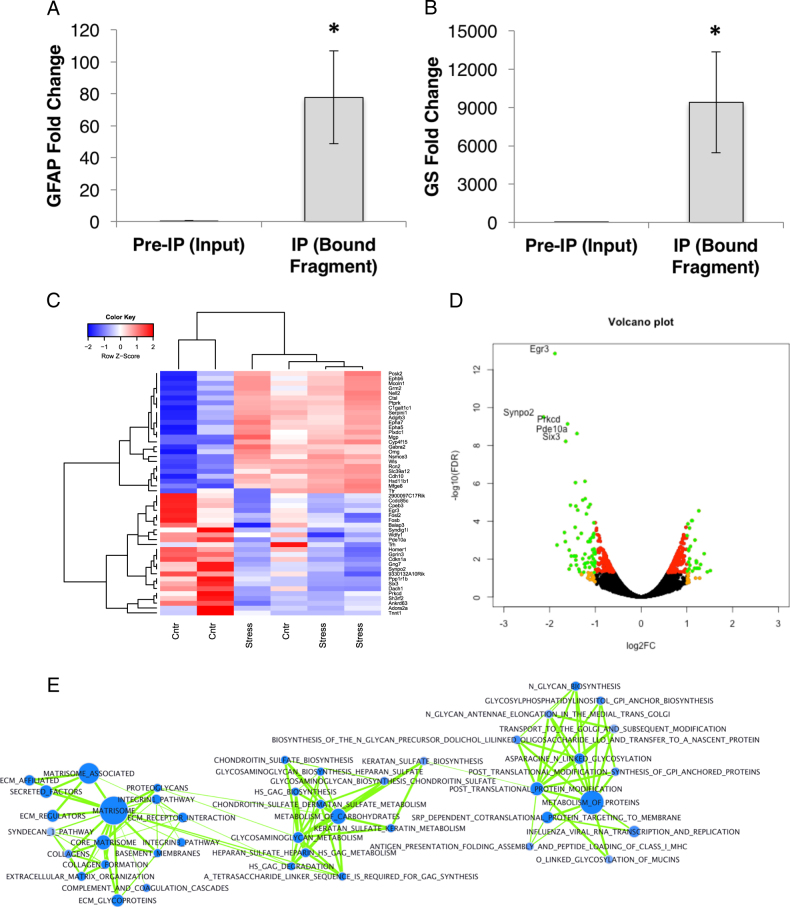
TRAPseq of astroglial translatome following CVS. **a**, **b** Fold change of GFAP and GS gene expression levels between input and immunoprecipitated TRAP fragments. **c** Heat map of the log-CPM values for the subset of top 25 upregulated and top 25 downregulated differentially expressed genes (genes were ranked by FDR corrected *p*-values). Red indicates high expression level and blue indicates low expression level. Samples and genes have been reordered by hierarchical clustering and a dendrogram is shown for both the sample and the gene clustering. **d** Volcano plot of the differentially expressed genes. Black: genes with no significant differential expression level; red: significantly differentially expressed genes at FDR < 0.05; orange: gene with abs(log2FC) > 1; green: significantly differentially expressed genes at FDR < 0.05 and abs(log2FC) > 1. Gene Symbols are highlighted for the green subset. Names of top five genes are shown. **e** Gene set network generated using the Cytoscape (v3.2.1) [CITE: http://doi:10.1101/gr.1239303] add-on EnrichmentMap [CITE: 10.1371/journal.pone.0013984]. Gene sets are represented as nodes (circles) in the network. Only significant (FDR < 0.05) C2 curated gene sets (canonical pathways) from the GSEA results are represented. Blue: upregulation in CVS; red: downregulation in CVS. Edge length is based on number of genes in common between two gene sets. Clustering of gene sets with similar function can be observed

In order to profile the astroglial translatome in response to stress, we performed TRAP to isolate cortical astroglial-specific translating transcripts followed by sequencing. To validate the enrichment of astroglial genes in our experiment, we used quantitative PCR to measure the levels of two astroglial genes, GS and GFAP, in the immunoprecipitated fragment (TRAP) compared to the total preimmunoprecipitation. We found significant elevations of both GS and GFAP, suggesting that our TRAPseq results were indeed based on an astroglial-specific translatome (Fig. 3a, b).

Differential gene expression analysis revealed that 545 genes were differentially expressed genes (DEGs) in response to CVS (Supplemental Data File DEG). Heat map and hierarchical clustering analysis showed that the gene expression profile of one control sample had either an upregulation or no change in genes that were typically upregulated in stress samples (Supplemental Fig. 1C). This may be a reflection of varying levels of baseline stress across individuals; nevertheless, to assess the impact of this sample’s contribution to our overall results, we conducted separate analyses including and excluding the sample and found no overall changes in our downstream analyses.

Analysis of DEGs in response to CVS showed enrichment in a wide array of canonical pathways and gene ontologies (GOs) (Supplemental Data File DEG ToppGene). Analysis of the DEGs with ToppGene Annotation (Supplemental Data File DEG ToppGene) revealed a mouse phenotype that was associated with abnormal affect/behavior, anxiety responses, and anxiety/fear behavior, validating the current DEG’s relationship both to our observed behavioral phenotype and to previous findings. Gene Set Enrichment Analysis (GSEA) [32] identified a number of stress-induced translational alterations in biologically relevant predefined groups of genes. GSEA revealed an upregulation of core extracellular matrix (ECM) signature (including ECM glycoproteins, collagens, and proteoglycans) and lysosome-related pathways in response to CVS. The upregulation of the ECM core and lysosome-related pathways suggests an increased production of ECM protein in response to CVS, in particular proteoglycans, and an increase in protein degradation, consistent with astroglial activation. These changes are particularly interesting because (1) astroglial cells are known to contribute to the organization and maintenance of PNNs, which are proteoglycan-rich ECM structures that surround cortical interneurons and are thought to be responsible for synaptic stabilization and inhibition of plasticity, therefore lending further support to a stress-induced decrease in astroglial-mediated plasticity [33–39]; and (2) because PNNs have recently been implicated in human psychiatric diseases associated with stress [37].

Consistent with the changes in canonical pathways, the top GO terms enriched with CVS involved lysosomes and ECM (see Supplemental data file GSEA Gene Ontology). Notably, top significantly upregulated genes were associated with p-glycoproteins which, when inhibited, facilitate antidepressant action [40]; ECM formation and maintenance (Sod3, Galns, Mgp, Lama4); and inflammatory pathways that are induced by lipopolysaccharide (LPS; which has been previously used to induce depressive-like behaviors), including Ptger3, Erap1, C1ra [41–43]; (Fig. 3d, Supplemental Data File DEG & Supplemental Fig. 1B). GSEA also highlighted a downregulation of overall transcription and calcineurin (CaN)-dependent nuclear factor of activated T cell (NFAT) signaling (Fig. 3e; see Supplemental data file GSEA Canonical Pathways). The downregulation of the CaN and NFAT pathways is particularly interesting as astroglial CaN is involved in astroglial glutamate uptake, the initiation of astroglial reactivity and inflammatory cascades, and is required for the resolution of inflammation [44]; which is consistent with a persistent inflammatory state of astroglia associated with exposure to CVS. Decreased CaN signaling results in dephosphorylation of NFAT and therefore NFAT targets such as GFAP should be decreased, which is consistent with our immunohistochemical observations. CaN is also important for synaptic plasticity [45] and the silencing of the CaN-NFAT pathways suggests decreased synaptic plasticity in stress. Previous studies in intact amyloid-bearing mice have shown that inhibition of astrocytic CaN-NFAT activity also results in a reduction in the surface area of individual hippocampal astrocytes, without altering the overall number of astrocytes [46], which may be consistent with decreased expression levels of the intermediate filament protein, GFAP. This may also explain the lower general transcriptional throughput highlighted by the GSEA analysis. The top downregulated genes include Synpo2 and EGR3, which are necessary for synaptic plasticity, long-term potentiation (LTP), learning and memory [47, 48]; Ptpn7, which belongs to a family of protein tyrosine phosphatases also necessary for synaptic plasticity and associated with models of developmental delay [49]; and Gprin3, part of the NMDA receptor complex which regulates the synaptic protein PSD-95 [50] (Fig. 3c, d, Supplemental Data File DEG and Supplemental Fig. 1C). Interestingly, we found an upregulation in Serpini1, which decreases PSD-95 expression, suggesting that altogether CVS decreased genes associated with synaptic plasticity [51].

Overall, the downregulated pathways point to an impairment or downregulation of signal transduction and transcription, possibly associated with a decrease in astroglial metabolism. This is more clearly represented by the GO terms enriched in downregulated genes (see Supplemental data file GSEA Gene Ontology). To further explore this transcriptional downregulation, we tested the gene sets corresponding to transcription factors and miRNAs and found that most of the listed gene sets upregulated in stress correspond to transcription factors and that only five gene sets, corresponding to three (known) transcription factors, were significantly enriched (See Supplemental data file GSEA MicroRNA and Transcription Factors). Conversely, the gene sets downregulated in stress show strong significance and correspond mostly to miRNAs, suggesting a strong active posttranscriptional (epigenetic) control of gene expression (see Supplemental data file GSEA GeneSet).

Gene sets that were downregulated in stress included miR302C and miR144, which are known tumor suppressors and associated with age-related decline [52–55], and members of the miR-181 family (see Supplemental data file GSEA GeneSet). Members of the miR-181 family have been shown to be expressed in astroglial cells and knockdown of these increased pro-inflammatory cytokines in response to LPS [56], suggesting an increased susceptibility to tumor formation and a persistent inflammatory state, which is consistent with the effects of chronic stress observed in both human and rodent models.

### PNNs mediate CVS-induced depressive-like behavior

**Fig. 4:**
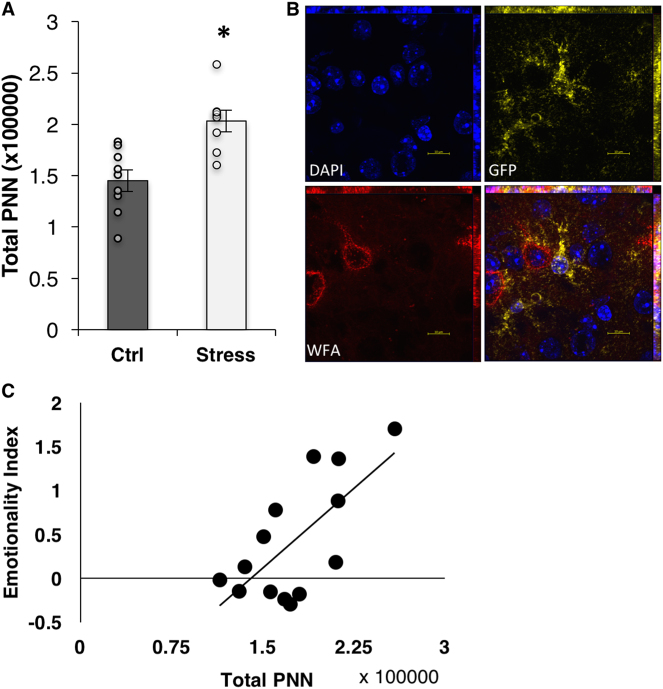
CVS induces an increase in cortical perineuronal nets. **a** Total number of cortical PNN+ cells per cortical hemisphere. **b** Representative confocal image (63×) showing co-localization of PNNs and astroglial processes (GFP). **c** Scatter plot showing the correlation between the number of PNN+ cells and the total emotionality score. Bars denote group mean and error bars denote SEM. *p = 0.001; data points reflect individual scores

Results from our astroglial TRAPseq showed significant changes in gene expression profiles related to ECMs and, in particular, PNNs. Astroglial cells have been previously shown to contribute to the fabrication, organization, and maintenance of PNNs [57]. While the role of PNNs has not been directly examined in the etiology of depressive- and anxiety-like behaviors, a number of postmortem studies in humans have suggested disturbances in cortical PNNs in psychiatric diseases such as depression, bipolar disorder, and schizophrenia, all of which include stress as a risk factor [37]. In order to validate CVS-induced PNN changes, we counted the number of cortical PNNs (using *Wisteria floribunda* lectin) and found a significant increase in the total number of PNNs in response to stress (*p* = 0.001; Fig. 4a). In addition, using high-magnification confocal imaging, we noted co-localization between astroglial processes and the PNNs (Fig. 4b).

Examination of the relationships between PNNs and key behavioral measures revealed a negative correlation between PNNs and the latency to immobility, *r* = −0.59, *p* = 0.01 (Table 1). Positive correlations were found between PNNs and the time immobile in the FST, *r* = 0.63, *p* = 0.008, and the total emotionality score, *r* = 0.69, *p* = 0.003 (Fig. 4c; Table 1).

To identify the best unique predictor of the latency to immobility in the FST, a multiple regression model with PNNs and Vimentin was conducted. The overall model was significant, *R* = 0.65, *R*^2^ = 0.32, *F* (3,10) = 4.02, *p* = 0.049; however, neither PNNs nor Vimentin accounted for a significant amount of unique variance when they were considered together in the model, *p* = 0.10 and *p* = 0.27, respectively. Similarly, a multiple regression was conducted with PNN and GFAP as predictors of total emotionality score, and the overall model was significant, *R* = 0.76, *R*^2^ = 0.49, *F* (3,10) = 7.35, *p* = 0.009. On examining the unique contributions of the predictors, only PNNs predicted the total emotionality score, *β* = 0.60*, t* = 2.96*, p* = 0.013, whereas GFAP was not significant, *p* = 0.14, reinforcing the potential for an important role for PNNs in the expression of depressive-like behaviors.

**Fig. 5:**
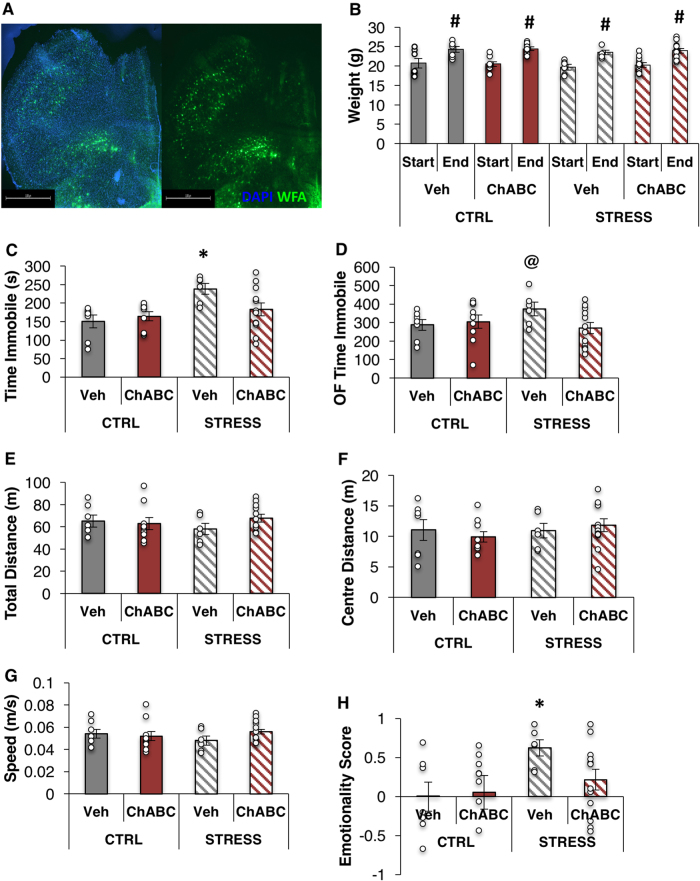
Perineuronal net degradation reverses CVS-induced depressive phenotype. **a** Photomicrograph of representative placement of cannula and location of infusion of ChABC. **b** Weight at the start and end of study. **c** Forced swim test: total time immobile in CTRL and CVS administered vehicle or ChABC bilaterally into the PFC. **d**–**g** Open field test: total immobility (**d**), total distance traveled in the entire field (**e**), distance traveled in center (**f**), and average speed (**g**). **h** Emotionality score [28]. Bars denote group mean and error bars denote SEM. *Group is significantly different from all other groups (*p* < 0.05); ^#^significant difference from start weight (*p* < 0.05); ^@^Stress, Veh group is significantly different from Ctrl, Veh and Stress, ChABC groups (*p* < 0.05); data points reflect individual scores

PNNs have been previously associated with decreased plasticity and a “cementing in” of learning and memory associated with the closure of critical periods. We therefore hypothesized that PNNs might conserve deleterious stress-induced changes in cortical networks and that degradation of the PNNs would reverse the effects of CVS. To achieve this, we subjected mice to 5 weeks of CVS and then injected ChABC, an enzyme which degrades PNNs, bilaterally into the PFC (Fig. 5a); ChABC-treated mice were otherwise healthy, showed a small increase in weight from the start to the end of the study, and showed no differences in weight from Vehicle-treated mice (Fig. 5b). Histological analysis of PNN number by counting WFA+ cells below the injection site showed a 37% decrease in PNNs in stressed mice that were injected with ChABC, bringing them down to control levels, and a 6% decrease in PNNs in control mice injected with ChABC. Degradation of PNNs induced a reversal of the stress-induced phenotype in the time immobile on the FST (Post-hoc Veh, Stress group different from all other groups (*p* < 0.05); no differences between all other groups (*p* > 0.05); Fig. 5c) and on the total time immobile of the open field test (Post-hoc Veh, Stress group different from CTRL, Veh and Stress, ChABC groups (*p* < 0.05); no differences between all other groups (*p* > 0.05); Fig. 5d). No effects of PNN degradation were observed on other measures of the open field, activity, and on the elevated plus maze (Fig. 5e–g and data not shown). Finally, we calculated the total emotionality score and we found a significant increase in emotionality by stress that was attenuated by ChABC treatment (Post-hoc: Veh, Stress group different from CTRL groups (*p* < 0.05), trend for difference from Stress, ChABC group (*p* = 0.06)). No changes in emotionality were noted with ChABC in non-stressed controls; however, this may be due to the lower levels of PNN degradation seen in this group.

## Discussion

In the current study, we examined the effects of a rodent model of depression on cortical astroglial cell expression of proteins associated with plastic states and related gene expression profiles. Although stress is an adaptive physiological reaction, chronic and uncontrollable stressors (such as job loss, death of a loved one, etc.) have been linked to both the onset of depression and exacerbation of symptoms. The use of the CVS paradigm employed in this study induced a reliable behavioral phenotype and allowed us to examine astroglial plasticity that may result from stress of a more chronic nature.

Several studies have shown a decrease in GFAP expression in cortical astroglial cells in postmortem tissue of depressed suicides and in animal models of depression, leading authors to conclude that there is a decrease in the total number of cortical astroglial cells in depressive-like phenotypes. Although we saw a similar decrease in both the total number and the proportion of GFAP+ cells in response to stress, we found no differences in total astroglia number when we assessed GFP. These findings caution interpretation of protein expression as a marker of total cell population number rather than specific protein expression levels. Moreover, because GFAP expression is upregulated in astroglial cells undergoing plastic change [58], this may be suggestive of a decrease in plasticity.

The notion that CVS induces decreased astroglial plasticity potential is further highlighted by the decreased expression of genes necessary for synaptic formation and LTP, lower transcriptional throughput, decreased growth factor expression and suppression of associated signaling pathways, and increases in genes associated with the formation and maintenance of ECMs and PNNs themselves. Taken together with previous observations of decreased cortical neuronal arborization in response to CVS [59], it would seem that this dampening of plasticity is not only neuronal specific, but given the central role of astroglia in controlling many (if not all) of these plastic events, they are likely primarily involved in mediating the effects of CVS on cortical neurons. The lower transcriptional throughput, together with the changes in transcription factors, microRNAs, and posttranslational modifications also suggest CVS-induced epigenetic changes. Indeed, many studies have demonstrated central epigenetic modifications induced by various stressors [60–63]; however, to our knowledge this has not been demonstrated within an astroglial cell population. Further studies will be needed to assess the relevance of epigenetic modifications within astroglial cells to the behavioral phenotype induced by CVS.

PNNs have been previously associated with the closure of critical periods and are thought to inhibit plasticity [39]. Increasing the potential for neural plasticity by degrading PNNs reversed some of the CVS-induced increases in depressive- and anxiety-like behaviors but not all. Importantly, most of the anxiety-like behaviors assessed were not impacted by degradation of PNNs. This may suggest a selective role of PNNs within the PFC on specific behaviors or it could also be a limitation of the time-point examined postinjection. FST is typically one of the most sensitive early markers of an antidepressant response and so future studies would need to assess long-term effects of prefrontal cortical PNN degradation on behavioral changes induced by chronic stress in order to conclusively determine whether PNN degradation is involved in depressive-like behaviors and not anxiety-like behaviors.

A growing body of literature has demonstrated important sex differences in the prevalence of mood and anxiety disorders, with females typically at higher risk [64, 65]. In addition, several subtypes of mood disorders are related to hormonal state such as premenstrual dysphoric disorder and postpartum depression, suggesting that ovarian hormone fluctuations may increase risk for mood and anxiety disorders or, conversely, that androgens play a protective role. Interestingly, astroglial cells show sexual dimorphism, express hormone receptors, and change in morphology and protein expression patterns in response to changes in hormonal and reproductive state [66–70]. Furthermore, cyclic changes in ovarian hormones have been shown to induce changes in astroglial-induced plasticity [71–73]. These findings lend further support to the potential crucial role for astroglia in the expression of depressive- and anxiety-like behaviors, now as key mediators of sex differences. The current study examined the effects of CVS on astroglial cells in male subjects only, therefore further studies are imperative to determine whether our findings are generalizable to female subjects.

Another important limitation in our study is the limited RNA yield obtained using the TRAP methodology, requiring pooled material and therefore preventing correlations of gene expression analysis with individual behavioral phenotypes. Immunohistochemical analysis of PNNs and several astroglial markers revealed some important co-relationships, for example, that the number of cortical PNNs were unique predictors of emotionality scores; however, future studies will be needed to assess the relationships between other astroglial gene expression changes and behavioral phenotypes. Similarly, because the entire neocortex was examined in the current study, further studies will be needed to examine the specific contributions of cortical subregions. Importantly, we used unbiased stereological sampling of PNNs and GFAP in response to CVS and sites did not show significant variability in expression patterns, as might be expected if expression levels varied greatly from one sampling site to another or across different regions of the neocortex.

Our analyses suggest that, altogether, CVS downregulates transcription factors and growth factor signaling in astroglia, concurrent with an upregulation in genes associated with protein degradation and PNNs. One major hurdle in developing novel pharmacotherapies for depression is to find rapid-acting drugs. Antidepressant medications take several weeks to reduce the depressive-like behaviors induced by CVS; however, promoting plasticity through the degradation of PNNs following stress induced a rapid reversal of the effects of CVS on depressive-like behavior. The current study, together with the growing body of literature on astroglia, stress, and depression, suggests that targeting cortical plasticity with drugs that modulate astroglial functions is a worthwhile avenue to explore in the development of next-generation antidepressants.

## Electronic supplementary material

Supplemental Methods

Supplemental Figure 1

Supp Data File DEG

Supp Data File DEGToppGene

Supp Data File GSEA Canonical Pathways

Supp Data File GSEA for GeneSet

Supp Data File GSEA Gene Ontology

Supp Data File MicroRNA X Transcription Factors

## References

[1] “Depression, A Global Public Health Concern” Developed by Marina Marcus, M. Taghi Yasamy, Mark van Ommeren, and Dan Chisholm, Shekhar Saxena WHO Department of Mental Health and Substance Abuse; http://www.who.int/mental_health/management/depression/who_paper_depression_wfmh_2012.pdf.

[2] Al-Harbi KS (2012). Treatment-resistant depression: therapeutic trends, challenges, and future directions. Patient Prefer Adherence.

[3] Krishnan V, Nestler EJ (2008). The molecular neurobiology of depression. Nature.

[4] McEwen BS, Eiland L, Hunter RG, Miller MM (2012). Stress and anxiety: structural plasticity and epigenetic regulation as a consequence of stress. Neuropharmacology.

[5] Czeh B, Di Benedetto B (2013). Antidepressants act directly on astrocytes: evidences and functional consequences. Eur Neuropsychopharmacol.

[6] Sofroniew MV, Vinters HV (2010). Astrocytes: biology and pathology. Acta Neuropathol.

[7] Silver J, Miller JH (2004). Regeneration beyond the glial scar. Nat Rev Neurosci.

[8] Nagy C, Suderman M, Yang J, Szyf M, Mechawar N, Ernst C (2015). Astrocytic abnormalities and global DNA methylation patterns in depression and suicide. Mol Psychiatry.

[9] Peng L, Verkhratsky A, Gu L, Li B (2015). Targeting astrocytes in major depression. Expert Rev Neurother.

[10] Rial D, Lemos C, Pinheiro H, Duarte JM, Goncalves FQ, Real JI (2015). Depression as a glial-based synaptic dysfunction. Front Cell Neurosci.

[11] Schwarz JM, Bilbo SD (2012). Sex, glia, and development: interactions in health and disease. Horm Behav.

[12] Smialowska M, Szewczyk B, Wozniak M, Wawrzak-Wlecial A, Domin H (2013). Glial degeneration as a model of depression. Pharmacol Rep.

[13] Banasr M, Duman RS (2008). Glial loss in the prefrontal cortex is sufficient to induce depressive-like behaviors. Biol Psychiatry.

[14] Cotter D, Mackay D, Chana G, Beasley C, Landau S, Everall IP (2002). Reduced neuronal size and glial cell density in area 9 of the dorsolateral prefrontal cortex in subjects with major depressive disorder. Cereb Cortex.

[15] Ongur D, Bechtholt AJ, Carlezon WA, Cohen BM (2014). Glial abnormalities in mood disorders. Harv Rev Psychiatry.

[16] Rajkowska G, Stockmeier CA (2013). Astrocyte pathology in major depressive disorder: insights from human postmortem brain tissue. Curr Drug Targets.

[17] Torres-Platas SG, Nagy C, Wakid M, Turecki G, Mechawar N (2016). Glial fibrillary acidic protein is differentially expressed across cortical and subcortical regions in healthy brains and downregulated in the thalamus and caudate nucleus of depressed suicides. Mol Psychiatry.

[18] Nagy C, Torres-Platas SG, Mechawar N, Turecki G (2016). Repression of astrocytic connexins in cortical and subcortical brain regions and prefrontal enrichment of H3K9me3 in depression and suicide. Int J Neuropsychopharmacol.

[19] Kajitani N, Hisaoka-Nakashima K, Morioka N, Okada-Tsuchioka M, Kaneko M, Kasai M (2012). Antidepressant acts on astrocytes leading to an increase in the expression of neurotrophic/growth factors: differential regulation of FGF-2 by noradrenaline. PLoS ONE.

[20] Dougherty JD, Schmidt EF, Nakajima M, Heintz N (2010). Analytical approaches to RNA profiling data for the identification of genes enriched in specific cells. Nucleic Acids Res.

[21] Doyle JP, Dougherty JD, Heiman M, Schmidt EF, Stevens TR, Ma G (2008). Application of a translational profiling approach for the comparative analysis of CNS cell types. Cell.

[22] Heiman M, Schaefer A, Gong S, Peterson JD, Day M, Ramsey KE (2008). A translational profiling approach for the molecular characterization of CNS cell types. Cell.

[23] Salmaso N, Silbereis J, Komitova M, Mitchell P, Chapman K, Ment LR (2012). Environmental enrichment increases the GFAP+ stem cell pool and reverses hypoxia-induced cognitive deficits in juvenile mice. J Neurosci.

[24] Salmaso N, Stevens HE, McNeill J, ElSayed M, Ren Q, Maragnoli ME (2016). Fibroblast growth factor 2 modulates hypothalamic pituitary axis activity and anxiety behavior through glucocorticoid receptors. Biol Psychiatry.

[25] Gundersen HJ, Bagger P, Bendtsen TF, Evans SM, Korbo L, Marcussen N (1988). The new stereological tools: disector, fractionator, nucleator, and point sampled intercepts and their use in pathological research and diagnosis. APMIS.

[26] West MJ (1993). New stereological methods for counting neurons. Neurobiol Aging.

[27] Kim JG, Suyama S, Koch M, Jin S, Argente-Arizon P, Argente J (2014). Leptin signaling in astrocytes regulates hypothalamic neuronal circuits and feeding. Nat Neurosci.

[28] Guilloux JP, Seney M, Edgar N, Sibille E (2011). Integrated behavioral z-scoring increases the sensitivity and reliability of behavioral phenotyping in mice: relevance to emotionality and sex. J Neurosci Methods.

[29] Rajkowska G, Miguel-Hidalgo JJ (2007). Gliogenesis and glial pathology in depression. CNS Neurol Disord Drug Targets.

[30] John CS, Sypek EI, Carlezon WA, Cohen BM, Ongur D, Bechtholt AJ (2015). Blockade of the GLT-1 transporter in the central nucleus of the amygdala induces both anxiety and depressive-like symptoms. Neuropsychopharmacology.

[31] Mayhew J, Beart PM, Walker FR (2015). Astrocyte and microglial control of glutamatergic signalling: a primer on understanding the disruptive role of chronic stress. J Neuroendocrinol.

[32] Subramanian A, Tamayo P, Mootha VK, Mukherjee S, Ebert BL, Gillette MA (2005). Gene set enrichment analysis: a knowledge-based approach for interpreting genome-wide expression profiles. Proc Natl Acad Sci USA.

[33] Dzyubenko E, Gottschling C, Faissner A (2016). Neuron-glia interactions in neural plasticity: contributions of neural extracellular matrix and perineuronal nets. Neural Plast.

[34] Faissner A, Pyka M, Geissler M, Sobik T, Frischknecht R, Gundelfinger ED (2010). Contributions of astrocytes to synapse formation and maturation—potential functions of the perisynaptic extracellular matrix. Brain Res Rev.

[35] Kim SY, Porter BE, Friedman A, Kaufer D (2016). A potential role for glia-derived extracellular matrix remodeling in postinjury epilepsy. J Neurosci Res.

[36] Song I, Dityatev A (2017). Crosstalk between glia, extracellular matrix and neurons. Brain Res Bull.

[37] Sorg BA, Berretta S, Blacktop JM, Fawcett JW, Kitagawa H, Kwok JC (2016). Casting a wide net: role of perineuronal nets in neural plasticity. J Neurosci.

[38] Viggiano D, Ibrahim M, Celio MR (2000). Relationship between glia and the perineuronal nets of extracellular matrix in the rat cerebral cortex: importance for volume transmission in the brain. Prog Brain Res.

[39] Carulli D, Kwok JC, Pizzorusso T (2016). Perineuronal nets and CNS plasticity and repair. Neural Plast.

[40] O’Brien FE, Dinan TG, Griffin BT, Cryan JF (2012). Interactions between antidepressants and P-glycoprotein at the blood-brain barrier: clinical significance of in vitro and in vivo findings. Br J Pharmacol.

[41] Goto Y, Ogawa K, Hattori A, Tsujimoto M (2011). Secretion of endoplasmic reticulum aminopeptidase 1 is involved in the activation of macrophages induced by lipopolysaccharide and interferon-gamma. J Biol Chem.

[42] Lazarus M (2006). The differential role of prostaglandin E2 receptors EP3 and EP4 in regulation of fever. Mol Nutr Food Res.

[43] Zamanian JL, Xu L, Foo LC, Nouri N, Zhou L, Giffard RG (2012). Genomic analysis of reactive astrogliosis. J Neurosci.

[44] Dmitry LFR, Mapelli L., Moccia F. (2016). From pathology to physiology of calcineurin signaling in astrocytes. Opera Med Et Physiol.

[45] Zeng H, Chattarji S, Barbarosie M, Rondi-Reig L, Philpot BD, Miyakawa T (2001). Forebrain-specific calcineurin knockout selectively impairs bidirectional synaptic plasticity and working/episodic-like memory. Cell.

[46] Furman JL, Sama DM, Gant JC, Beckett TL, Murphy MP, Bachstetter AD (2012). Targeting astrocytes ameliorates neurologic changes in a mouse model of Alzheimer’s disease. J Neurosci.

[47] Deller T, Korte M, Chabanis S, Drakew A, Schwegler H, Stefani GG (2003). Synaptopodin-deficient mice lack a spine apparatus and show deficits in synaptic plasticity. Proc Natl Acad Sci USA.

[48] Li L, Yun SH, Keblesh J, Trommer BL, Xiong H, Radulovic J (2007). Egr3, a synaptic activity regulated transcription factor that is essential for learning and memory. Mol Cell Neurosci.

[49] Kamceva M, Benedict J, Nairn AC, Lombroso PJ (2016). Role of striatal-enriched tyrosine phosphatase in neuronal function. Neural Plast.

[50] Tu S, Shin Y, Zago WM, States BA, Eroshkin A, Lipton SA (2007). Takusan: a large gene family that regulates synaptic activity. Neuron.

[51] Tsang VW, Young D, During MJ, Birch NP (2014). AAV-mediated overexpression of neuroserpin in the hippocampus decreases PSD-95 expression but does not affect hippocampal-dependent learning and memory. PLoS ONE.

[52] Huang J, Shi Y, Li H, Yang M, Liu G (2015). MicroRNA-144 acts as a tumor suppressor by targeting Rho-associated coiled-coil containing protein kinase 1 in osteosarcoma cells. Mol Med Rep.

[53] Kuo CH, Deng JH, Deng Q, Ying SY (2012). A novel role of miR-302/367 in reprogramming. Biochem Biophys Res Commun.

[54] Pi J, Tao T, Zhuang T, Sun H, Chen X, Liu J (2017). A microRNA302-367-Erk1/2-Klf2-S1pr1 pathway prevents tumor growth via restricting angiogenesis and improving vascular stability. Circ Res.

[55] Szafranski K, Abraham KJ, Mekhail K (2015). Non-coding RNA in neural function, disease, and aging. Front Genet.

[56] Hutchison ER, Kawamoto EM, Taub DD, Lal A, Abdelmohsen K, Zhang Y (2013). Evidence for miR-181 involvement in neuroinflammatory responses of astrocytes. Glia.

[57] Wiese S, Karus M, Faissner A (2012). Astrocytes as a source for extracellular matrix molecules and cytokines. Front Pharmacol.

[58] Walz W (2000). Controversy surrounding the existence of discrete functional classes of astrocytes in adult gray matter. Glia.

[59] Liston C, Miller MM, Goldwater DS, Radley JJ, Rocher AB, Hof PR (2006). Stress-induced alterations in prefrontal cortical dendritic morphology predict selective impairments in perceptual attentional set-shifting. J Neurosci.

[60] Meaney MJ, Szyf M (2005). Environmental programming of stress responses through DNA methylation: life at the interface between a dynamic environment and a fixed genome. Dialogues Clin Neurosci.

[61] Anacker C, O’Donnell KJ, Meaney MJ (2014). Early life adversity and the epigenetic programming of hypothalamic-pituitary-adrenal function. Dialogues Clin Neurosci.

[62] Anisman H, Merali Z, Stead JD (2008). Experiential and genetic contributions to depressive- and anxiety-like disorders: clinical and experimental studies. Neurosci Biobehav Rev.

[63] Buschdorf JP, Meaney MJ (2015). Epigenetics/programming in the HPA axis. Compr Physiol.

[64] Gorman JM (2006). Gender differences in depression and response to psychotropic medication. Gend Med.

[65] Altemus M, Sarvaiya N, Neill Epperson C (2014). Sex differences in anxiety and depression clinical perspectives. Front Neuroendocrinol.

[66] Acaz-Fonseca E, Avila-Rodriguez M, Garcia-Segura LM, Barreto GE (2016). Regulation of astroglia by gonadal steroid hormones under physiological and pathological conditions. Prog Neurobiol.

[67] Garcia-Segura LM, Naftolin F, Hutchison JB, Azcoitia I, Chowen JA (1999). Role of astroglia in estrogen regulation of synaptic plasticity and brain repair. J Neurobiol.

[68] Salmaso N, Cossette MP, Woodside B (2011). Pregnancy and maternal behavior induce changes in glia, glutamate and its metabolism within the cingulate cortex. PLoS ONE.

[69] Salmaso N, Nadeau J, Woodside B (2009). Steroid hormones and maternal experience interact to induce glial plasticity in the cingulate cortex. Eur J Neurosci.

[70] Salmaso N, Popeski N, Peronace LA, Woodside B (2005). Differential effects of reproductive and hormonal state on basic fibroblast growth factor and glial fibrillary acid protein immunoreactivity in the hypothalamus and cingulate cortex of female rats. Neuroscience.

[71] Amateau SK, McCarthy MM (2002). Sexual differentiation of astrocyte morphology in the developing rat preoptic area. J Neuroendocrinol.

[72] McCarthy MM, Todd BJ, Amateau SK (2003). Estradiol modulation of astrocytes and the establishment of sex differences in the brain. Ann NY Acad Sci.

[73] Salmaso N, Woodside B (2008). Fluctuations in astrocytic basic fibroblast growth factor in the cingulate cortex of cycling, ovariectomized and postpartum animals. Neuroscience.

