# Supplementary material for: Profiling changes in cortical astroglial cells following chronic stress
Source: Neuropsychopharmacology. 2018 May 29;43(9):1961–71. doi: 10.1038/s41386-018-0105-x (PMC6046043; doi:10.1038/s41386-018-0105-x)
Supplement: Supplementary file 1 — Supplemental Methods [file 41386_2018_105_MOESM1_ESM.docx]

***Supplemental Methods:***

***CVS:*** Stressors included: altered light/dark cycle, food and water deprivation (overnight), isolation, cage tilt (30 degrees), exposure to odor, soiled bedding (~200 ml water added to bedding material), no bedding, swim for 10 minutes and lastly, restraint (for 15 minutes, in restraint cones that have an opening in the front for ventilation). The stressors were applied in a random and variable order, for various durations (up to a maximum in accordance with the protocol) *(see Figure 1A for timeline and stress schedule)*. Animals were monitored both during and immediately after each procedure and continuously during both the restraint and swimming procedures. They were observed for general condition, decreased locomotor activity or lethargy, decreased grooming and other unusual behaviors judged consistent with distress. Animals in the control group were housed in a separate room of similar size and environmental conditions (including temperature, food, water, light and humidity, etc.) but were simply not exposed to the experimental stressors.

***Surgical Procedures:*** ChABC (Chondoitinase ABC from proteus vulgaris lyophilized powder 0.3-3units/mg- Sigma-Aldrich ) was made fresh daily prior to surgeries by diluting in sterile saline solution at a concentration of 5 mU/μl. Meloxicam SR (4mg/kg) (Chiron, USA) was administered via sub cutaneous injection immediately prior to each surgery to provide sustained analgesia without the need for repeated injection stress. Mice underwent stereotaxic surgery using isofluorane (PPC, Canada) maintained at a rate of 1.5-2% throughout the procedure. A bilateral guide cannula, 360 um in internal diameter (Plastics One, Canada), was aimed at the PFC: from Bregma coordinates were (A/P 2.0mm, D/V -1.0mm, M/L 0.3mm (bilateral guide cannula 0.6mm inner diameter to inner diameter)). ChABC was delivered into the brain by a bilateral injector introduced through the guide cannula protruding 0.5 mm from the tip of the guide cannula. A 1 μl injection of either ChABC (5 mU/μl), diluted in saline, or vehicle (saline) was infused at a rate of 0.1 ul per minute, followed by a 5-minute diffusion period. After 5 minutes, the cannula was removed, bone wax (Ethicon USA) was used to fill the cranial injection site and the incision was closed using silk sutures (Ethicon USA). Animals were monitored immediately after each procedure, followed by daily monitoring. No adverse effects were noted, all mice were healthy and recovered quickly.

Following four days of recovery from the procedure, mice were tested on depressive- and anxiety-like behaviors from least to most stressful. Twenty-four hours following the last behavioral test, mice were perfused and brains harvested for immunocytochemical analysis of injection placement, 10 mice in total (across all groups) were removed from the final analysis due to faulty injection placement (Refer to supplemental methods Table A for final individual group numbers).

Behavioral Tests:

***Open Field Test***- Mice were placed in the corner of a brightly lit box (50 x 50cm x 35cm) and videotaped with AnyMaze Video Tracking System for 5 (CVS behavior data) or 20 minutes (CVS behavior data after PFC injection) (Salmaso *et al*, 2016). Time spent in the pre-defined zones (periphery and center) was recorded. Anxiety-like and exploratory behaviors were measured and included the amount of time an animal spent in a given zone, as well as motor activity.

***Elevated Plus Maze***- Mice were each placed in the center of the maze, facing an open arm (Salmaso *et al*, 2016). The dimensions of these arms are 30 x 5cm, with two enclosed with 25cm walls, and elevated 30cm from the floor. The mouse was then recorded exploring the maze for a total of 5 minutes, using AnyMaze Video Tracking System. The amount of time spent in the open and closed arms was recorded.

***Forced Swim Test***- In order to assess depressive-like effects that may be induced by CVS (e.g., learned helplessness), the forced swim test was used as per our previous studies (Salmaso *et al*, 2016). Time immobile, latency to become immobile and time spent swimming were recorded on a video camera and scored by an independent observer blind to the conditions of the experiment.

***Emotionality Score-*** The emotionality score was derived as previously described in Guilloux (2011). Z scores were calculated and normalized for locomotor behavior as appropriate therefore:

Z score OF=

$$\frac{\left( \frac{X-\mu}{\sigma} \right)TimeCenter+\left( \frac{X-\mu}{\sigma} \right)Proportion Peripheral Distance}{2}$$

Z score EPM=

$$\frac{\left( \frac{X-\mu}{\sigma} \right)TimeOpen+\left( \frac{X-\mu}{\sigma} \right)Proportion Closed Distance}{2}$$

Z score FST=

$$\left( \frac{X-\mu}{\sigma} \right)TIme Immobile$$

Emotionality score=

$$\frac{ZscoreOF+ZscoreEPM+ZscoreFST}{3}$$

***RNA Seq Library Prep*:** mRNA was purified from approximately 500ng of total RNA with oligo-dT beads and sheared by incubation at 94C. Following first-strand synthesis with random primers, second strand synthesis was performed with dUTP for generating strand-specific sequencing libraries. The cDNA library was then end-repaired and A-tailed, adapters were ligated and second-strand digestion was performed by Uricil-DNA-Glycosylase. Indexed libraries that met appropriate cut-offs for both were quantified by qRT-PCR using a commercially available kit: Kapa Library Quant Kit (Illumina) (KAPA Biosystems # KK4854-07960298001) and insert size distribution was determined with the LabChip GX or Agilent Bioanalyzer. Only samples with a yield of ≥0.5 ng/ul were used for sequencing.

***Flow Cell Preparation and Sequencing*:** Sample concentrations were normalized to 10 nM and loaded onto Illumina Rapid or High-output flow cells at a concentration that yields 130-250 million passing filter clusters per lane. Samples were sequenced using 75 bp paired-end sequencing on an Illumina HiSeq 2500 according to Illumina protocols. The 6 bp index was read during an additional sequencing read that automatically follows the completion of read 1. Data generated during sequencing runs were simultaneously transferred to the YCGA high-performance computing cluster. A positive control (prepared bacteriophage Phi X library) provided by Illumina is spiked into every lane at a concentration of 0.3% to monitor sequencing quality in real time.

***TRAPseq Data Analyses***

The RNAseq reads were mapped using Tophat (ver 2.2.1)(Kim *et al*, 2013) and Bowtie (ver 2.2.9)(Langmead and Salzberg, 2012) the Mus Musculus reference genome release GRCm 38 (p5) and the Gencode reference transcriptome vM13. The mapping rates ranged from 74% to 92.8%. The expression levels of the 50602 annotated genes were quantified using featureCounts(Liao *et al*, 2014) with the following options: -t exon; -g gene_id; -F “GTF”; -B; -s

0; -p; -a. This resulted in a number of ‘assigned’ reads from 22 million up to 30 million, with the exception of sample NS3 (CVS sample), which produced 14 million assigned reads (see Table QC in Supplemental Figure 1 for more details).

Raw read counts were filtered requiring more than 1 CPM (counts per million) in at least 3 samples and 14525 survived the filter and were considered for further analysis. Conditional Quantile Normalization (cqn package ver 1.16.0)(Kasper D. Hansen, 2012) was used to normalize counts and generate normalized RPKM. Hierarchical clustering (using all the expressed genes) shows the sample-to-sample relationship and relative clustering (see Supplemental Figure 1B).

Differentially expressed genes (DEGs) were inferred using the package edgeR (ver 3.12.1)(McCarthy *et al*, 2012) using the trended dispersion and the GLM capability of the package. P-values were adjusted for multiple testing using the Benjamini-Hochberg procedure. Corrected p-values less than 0.05 were considered statistically significant. FastQC v0.10.1 and RNA-SeQC (v1.1.8)(DeLuca *et al*, 2012) were used for QC (see Supplemental Figure 1A). Functional annotation was done using the ToppFun functionality of the ToppGene Suite(Chen *et al*, 2009).

The expression levels of the 14525 genes in all samples was used as input to Gene Set Enrichment Analysis (GSEA)(Subramanian *et al*, 2005), v3.0, and the C2, C3 and C5 gene sets of the Molecular Signatures Database v6.0 were tested for enrichment. GSEA was run with default parameters using the default genes ranking by Signal2Noise. FDR corrected p-values less than 0.05 were considered statistically significant.

**Supplemental Table A- Number of subject/experiment**

| **GROUP** | **EXPERIMENT** |  |  |  |
| --- | --- | --- | --- | --- |
|  | **Behavior** | **Sterelogical Cell Counting** | **TRAP-RNAseq** | **PNN Behavior** |
| **CONTROL** | 18 | 9 Total  (4 GFP+,  5 GFP-) | 6 (2 brains pooled) GFP + | 22 Pre-surgical  Final after histology:  7 VEH, 9 chABC |
| **STRESS** | 18 | 8 Total   (5 GFP+,  3 GFP-) | 6 (2 brains pooled) GFP + | 22 Pre-surgical  Final after histology:  6 VEH, 12 chABC |

**Supplemental Table B- List of antibodies used**

**
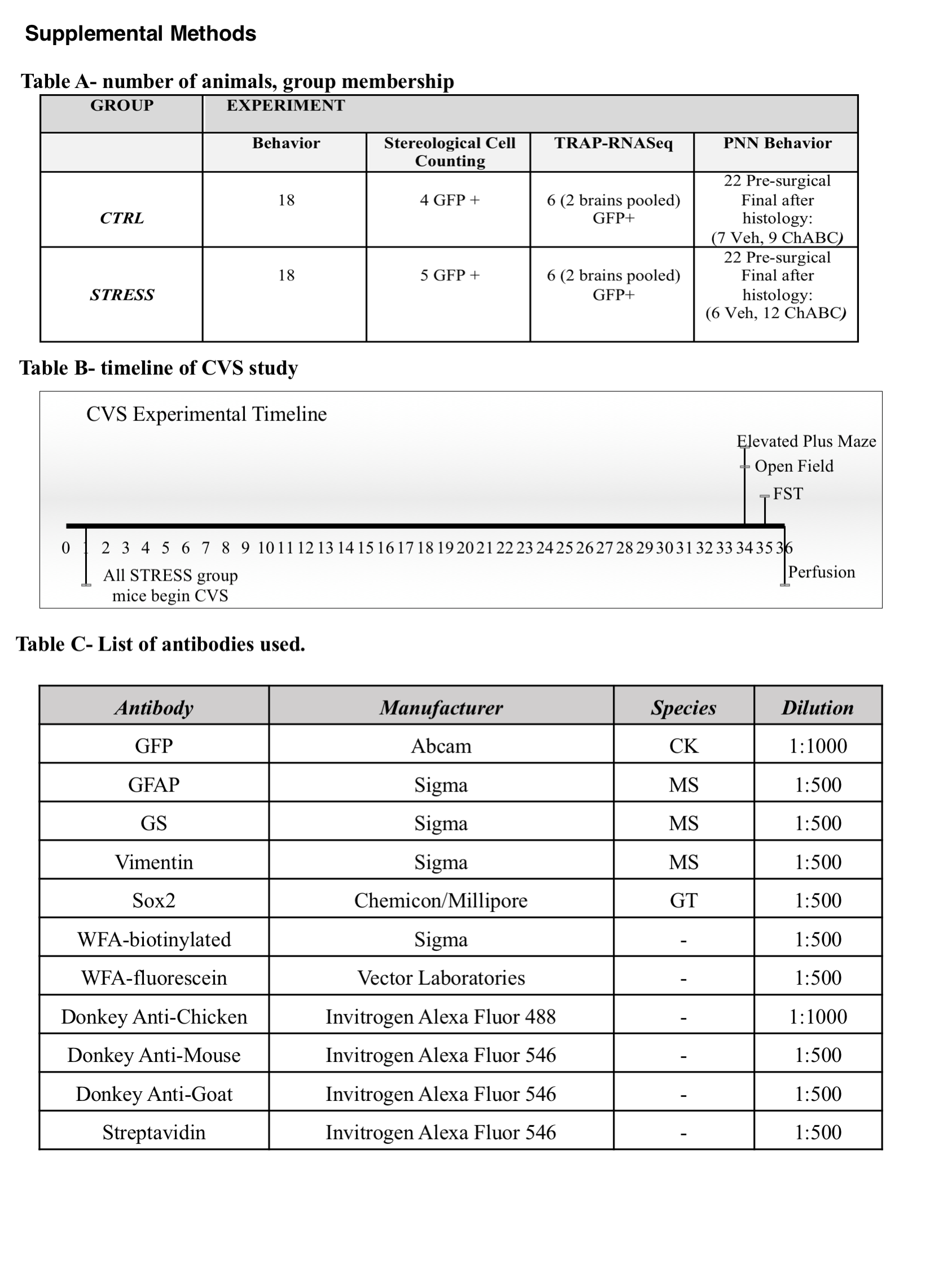
**

**Supplemental Table C- List of Stressors**

| **DAY** | **STRESSORS** | **DURATION** |
| --- | --- | --- |
| 1 | Cage tilt, wet bedding | 4hrs, overnight |
| 2 | Restraint, lights on | 15min, overnight |
| 3 | Odour, Swim | 5hrs, 10min |
| 4 | Cage tilt, empty cage | 2hrs, overnight |
| 5 | Wet bedding, lights on | 4hrs, overnight |
| 6 | Restraint, odour | 15min, 4hrs |
| 7 | Cage tilt, lights on | 5hrs, overnight |
| 8 | Odour, empty cage | 3hrs, overnight |
| 9 | Restraint, cage tilt | 15min, 3hrs |
| 10 | Swim, lights on | 10min, overnight |
| 11 | Odour, wet bedding | 3hrs, overnight |
| 12 | Cage tilt, empty cage | 4hrs, overnight |
| 13 | Cage tilt, lights on | 5hrs, overnight |
| 14 | Restraint, odour | 15min, 4hrs |
| 15 | Swim, wet bedding | 10min, overnight |
| 16 | Cage tilt, empty cage | 3hrs, overnight |
| 17 | Restraint, odour | 15min, 5hrs |
| 18 | Swim, lights on | 10min, overnight |
| 19 | Wet bedding, odour | 3hrs, 3hrs |
| 20 | Cage tilt, empty cage | 3hrs, overnight |
| 21 | Restraint, lights on | 15min, overnight |
| 22 | Cage tilt, odour | 3hrs, 4hrs |
| 23 | Wet bedding, empty cage | 3hrs, overnight |
| 24 | Restraint, wet bedding | 15min, 4hrs |
| 25 | Odour, lights on | 4hrs, overnight |
| 26 | Cage tilt, odour | 3hrs, 3hrs |
| 27 | Wet bedding, restraint | 4hrs, 15min |
| 28 | Odour, empty cage | 5hrs, overnight |
| 29 | Cage tilt, wet bedding | 4hrs, 3hrs |
| 30 | Restraint, lights on | 15min, overnight |
| 31 | Cage tilt, wet bedding | 3hrs, 4hrs |
| 32 | Odour, lights on | 4hrs, overnight |
| 33 | Restraint, cage tilt | 15min, 4hrs |
| 34 | Surgery | - |
| 35 | Surgery | - |
