# Supplementary figures and images for: Profiling changes in cortical astroglial cells following chronic stress
Source: Neuropsychopharmacology. 2018 May 29;43(9):1961–71. doi: 10.1038/s41386-018-0105-x (PMC6046043; doi:10.1038/s41386-018-0105-x)

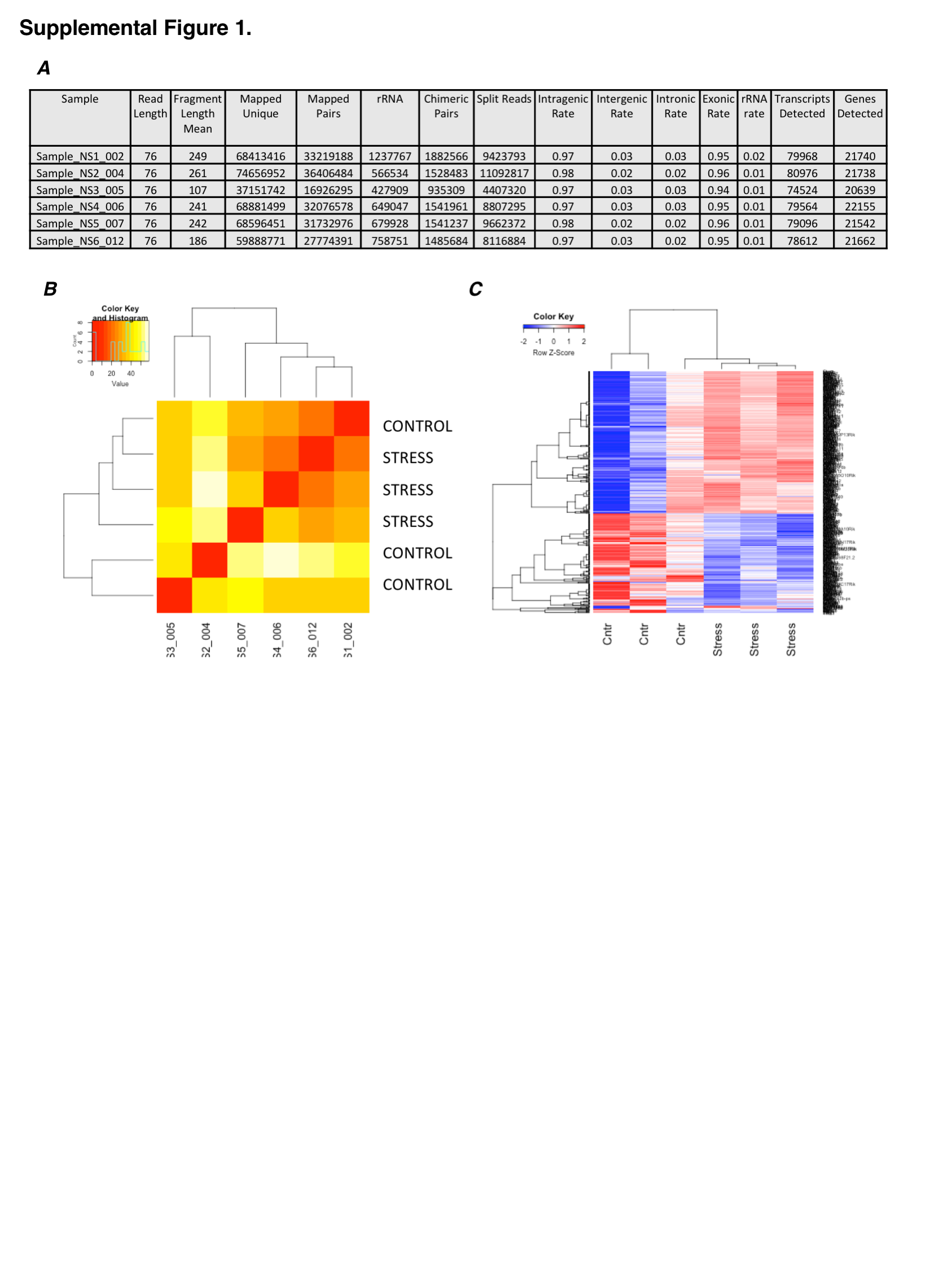

Supplement: Supplementary file 2 — Supplemental Figure 1 [file 41386_2018_105_MOESM2_ESM.tif]
